# Impact of busulfan pharmacokinetics on outcome in adult patients receiving an allogeneic hematopoietic cell transplantation

**DOI:** 10.1038/s41409-022-01641-6

**Published:** 2022-03-31

**Authors:** Claire Seydoux, Raphael Battegay, Joerg Halter, Dominik Heim, Katharina M. Rentsch, Jakob R. Passweg, Michael Medinger

**Affiliations:** 1 Division of Hematology, University Hospital of Basel, Basel, Switzerland; 2 Department of Laboratory Medicine, University Hospital of Basel, Basel, Switzerland

**Keywords:** Stem-cell research, Haematological cancer

## Abstract

Busulfan (Bu) is widely used in conditioning regimens before allogeneic hematopoietic cell transplantation, with variable metabolism due to interindividual differences of pharmacokinetics (PK). The purpose of this study was to correlate pharmacokinetics and clinical outcomes. Lower-AUC, in range-AUC and higher-AUC were defined as ±25% of the targeted Bu-AUC. In 2019, we changed Bu dosing from 4×/day (Bu-4) to 1×/day (Bu-1) for ease of application. AUC-target range was reached in 46% of patients; 40% were in low-AUC and 14% in high-AUC. Among all toxicities, viral and fungal infections were significantly more frequent in high-AUC compared with low-AUC (20% vs. 8%; *p* = 0.01 and 37% vs. 17%; *p* = 0.03). Bu-1 showed lower PK values (66% vs. 36% of Bu-4 in low-AUC; *p* < 0.01) and higher incidence of mucositis (*p* = 0.02). Long-term outcomes at 2 years showed a higher non-relapse mortality (NRM) (*p* < 0.01) and higher relative risk of death in the high-AUC group compared to the other groups. Cumulative incidence of relapse and acute/chronic GvHD were not significantly different. The optimal cut-off in Bu-AUC associated with low NRM was 969 µmol/l*min (ROC AUC 0.67, sensitivity 0.86 and specificity 0.47) for Bu-4. In conclusion, low-AUC BU-PK seems of benefit regarding NRM and survival.

## Introduction

Busulfan (Bu) is a widely used conditioning regimen before allogeneic hematopoietic stem cell transplantation (allo-HCT) to treat hematological diseases [1]. It is either used as myeloablative conditioning when combined with cyclophosphamide (BuCy or CyBu), thiopeta-fludarabine (TBF) or with fludarabine alone (FluBu4; 4 days busulfan) or as reduced-intensity conditioning (RIC) when given with a reduced dosing with 2 days of busulfan (FluBu2). The maximal tolerated amount of the drug is limited by liver injury; the most feared hepatic complication being the sinusoidal obstructive syndrome (SOS); formerly veno-occlusive disease (VOD) [2, 3]. As Bu has a narrow therapeutic index and its metabolism is variable among the population, the interindividual variability of the pharmacokinetics has an impact on toxicity and outcome [4–6]. Hypothesis behind this pharmacokinetic differences is that Bu is metabolized by hepatic enzymes such as Glutathione-S-Transferase and cytochrome P450 enzymes; pathways which are highly influenced by the pharmakogenetic diversity of patients [7–13]. To reduce toxicity of the drug, Bu has been given intravenously since the 2000’s, as oral administration shows a higher rate of SOS with a rather unpredictable variability in metabolism [14–16]. Bu pharmacokinetics (Bu-PK) after intravenous Bu administration is performed since 2013 in Basel as standard therapeutic drug monitoring (TDM), permitting a dose individualization for each patient according to the calculated area-under-the-curve (AUC) [17]. TDM has permitted to decrease mainly hepatic toxicity and especially the incidence of SOS, which plays an important role in non-relapse mortality (NRM) [4, 18]. Since April 2019, Bu infusion was switched from 4 times daily (Bu-4) to once daily (Bu-1) in Basel, as this is more comfortable for application without showing an increase prevalence in toxicity or end-organ damage [19, 20]. Bu-AUC target values are still controversial among studies, the target range used in Basel is 900–1350 µmol/l*min for Bu-4 and of 4680–5848 µmol/l*min for Bu-1, permitting a good balance between myeloablation and toxicity.

With this retrospective study, the correlation between Bu dosing and the proportion of patients who are in the Bu-PK target range (AUC) was studied. Secondary organ toxicities and long-term outcomes in patients receiving Bu as part as their conditioning regimen was determined, as well as the impact on Bu-PK and toxicity when Bu is given once versus four times daily (at same dose).

## Patients and methods

This is a retrospective study done at the University Hospital of Basel (Switzerland) from January 2013 to December 2020, comparing the relation between Bu dosing, Bu-PK and clinical outcome in adult patients receiving a Bu-conditioning regimen followed by allo-HCT. From January 2013 until March 2019, Bu was given four times daily (0.8 mg/kg) and was switched to once daily (3.2 mg/kg) since April 2019 (at same daily dose). The study was approved by the local ethics committee (EKNZ 2020–02782). The primary endpoints were the relation between Bu dose and the proportion of patients reaching the target Bu-PK AUC and furthermore all short-term treatment-related toxicities at hospital discharge of the patient (hepatic toxicity, mucositis, infectious complications, renal impairment, neurological involvement, pulmonary or cardiac dysfunction, dermatological toxicity, multi organ failure (MOF) and acute graft-versus-host-disease). Secondary endpoints were the relation of long-term outcomes to Bu-PK, namely chronic GvHD, NRM, relapse, GvHD-free-relapse-free survival, overall mortality and causes of death, as well as the relation between Bu-4 or Bu-1 with Bu-PK and the possible impact on toxicities mentioned above.

We included all consecutive adult patients with myeloid malignancies such as acute myeloid leukemia (AML), myelodysplastic syndrome (MDS) or myeloproliferative neoplasia (MPN) and chronic myeloid leukemia (CML) registered in the EBMT database of the University of Basel from 2013 to 2020, who received a Bu-conditioning regimen and in whom Bu-PK was performed. We recorded patient and donor baseline characteristics (sex, age, type and stage of disease, conditioning regimen, type of donor, prior HCT, stem cell source, Bu-PK) as well as follow-up up to 6 years after transplant.

### Treatment

The patients received conditioning regimens according to underlying disease, disease stage, age and co-morbidities, donor type and HLA-match followed by allo-HCT. Bu was given intravenously with a dose of 4 × 0.8 mg/kg over 2 h in Bu-4 and 3.2 mg/kg over 3 h with Bu-1. In young patients (<55 years) with few co-morbidities, CyBu or BuCy regimen was preferred with Bu given for 4 days, followed or preceded by i.v. Cyclophosphamide for 2 days as published [21], with a time interval >24 h between the two drugs [22]. TBF was given to patients with HLA haploidentical or high-risk one-antigen mismatched unrelated donors, consisting of Thiotepa 2 days, followed by Bu and Fludarabine for 2 days. We used myeloablative FluBu4 (4 days Bu, 5 days Flu) in patients not fit enough for CyBu or BuCy or non-myeloablative FluBu2 (2 days Bu, 5 days Flu) in patients with advanced age or relevant co-morbidities. An description of the chemotherapeutic protocols is displayed in Supplementary Table 1.

Graft-versus-host disease (GvHD) prophylaxis administered along with conditioning was cyclosporine A (CsA) and methotrexate as well as anti-T-cell globulins (ATG) in case of an unrelated donor. In matched related donors ≥40 years, GvHD prophylaxis was performed using ATG [23]. In patients with TBF conditioning, GvHD prophylaxis consisted of cyclophosphamide (50 mg/kg on day +3 and +4 in patients with a haploidentical donor or 40 mg/kg in patients with a one-antigen mismatched unrelated donor), and CsA and mycophenolate mofetil (MMF) starting on day +5. All patients received a SOS prophylaxis, consisting of heparin i.v. 5000 IU/day and ursodeoxycholic acid 250 mg 3×/day until neutrophil engraftment.

### BUsulfan pharmacokinetics

Bu plasma concentrations were determined by LC–MS/MS in heparinated plasma samples at 5 respectively 6 different time points after start of the first infusion (Bu-1: 3, 3.25, 4.5, 6, 8 and 11 h; Bu-4: 2, 2.5, 3, 4 and 6 h). The 11 h value in Bu-1 was omitted in the course, as it did not have significant impact on the calculated area-under-the-curve (Bu-AUC). The resulting AUC and concentration at steady state (Css) were calculated using the trapezoidal rule and permit to deduce Bu clearance (Bu/AUC). The target values for the Bu-AUC were 900–1350 µmol/l*min for Bu-4 and of 4680–5848 µmol/l*min for Bu-1 [21, 24]; Bu clearance normal range comprised clearance from 2.1 to 3.5 ml/min/kg [25]. This AUC-target range was set to assure myeloablation and engraftment without increasing the risk of serious treatment-related toxicities [23, 26]. Dose adjustment was realized when the Bu-AUC was ±25% over or under the Bu-AUC target range. The daily Bu dose was adjusted with a 25% increase or decrease of the dose; exception were patients with clinical relevant hepatic toxicity at time of Bu dosing (i.e., ASAT and ALAT > 100 U/l, bilirubin ≥ 34 µmol/l), in whom no adjustment was performed because of increased risk of SOS occurrence. The population was separated in three groups according to their first AUC value, namely patients under the target range (low-AUC, Bu- AUC < 900 µmol/l*min for Bu-1 and <4680 µmol/l*min for Bu-4), in range and above the target range (high-AUC, Bu-AUC > 1350 µmol/l*min for Bu-1 and >5848 µmol/l*min for Bu-4). For practical reasons, Bu dose was adjusted by a 25% dose increase or 25% dose reduction.

### Definitions

*Mucositis* was defined as clinically relevant with a grade ≥ III, most of the patients requiring parenteral nutrition for at least 3 days and/or continuous intravenously pain relief (morphine or hydromorphone) (NCI Common Terminology Criteria for Adverse Events (CTCAE version 4.0)). *SOS/VOD* was defined with the occurrence ≥2 of the followings criteria: hyperbilirubinemia >34 µmol/L [2 mg/dL], hepatomegaly or right upper quadrant pain of liver origin, and sudden weight gain (>*2%* of baseline body weight) because of fluid accumulation (modified Seattle criteria [27]. *Other organ toxicities* included the following: hepatic (hepatic failure with transaminase perturbation with ASAT and ALAT > 100 U/l, bilirubin ≥34 µmol/l), cardiac (cardiac decompensation, arrhythmia, toxic cardiomyopathy), pulmonary (pulmonary insufficiency requiring non-invasive or invasive ventilation, alveolar hemorrhage, acute respiratory distress syndrome), uro-renal (renal insufficiency with creatinine > 2.5× baseline, hemorrhagic cystitis), neurologic (seizure, acute confusional state, stroke, intra-cerebral hemorrhage), dermatologic (dermatitis, erythema, keratitis sicca) and MOF (combination of ≥3 of the above, dermatologic exlcuded). *Infectious complications* consisted of any laboratory/microbiological/clinical proven viral, bacterial or fungal infection, either in blood culture, biopsy or blood replication, including HHV-6, EBV or CMV replication and BK cystitis. *GvHD* grade and stage were classified using international guidelines for acute and chronic GvHD [28, 29]; acute GvHD was defined as clinically relevant with grade ≥ II.

### Statistical analysis

We reported data as mean or median with its standard deviation (SD) or with interquartile range (IQR) according to type of variable. Univariate analysis consisted of Chi-square-test for categorical variables, and Student’s *t*- or Mann–Whitney *U* test for continuous variables. Cumulative incidences were analyzed using Gray’s test. Significant variables in univariate analysis were entered into multivariate regression models. Long-term outcomes with survival curves were calculated using Kaplan-Meier model. Competing risk regression were used in multivariate analysis models. Because differences in Bu-AUC were hypothesized to exist between patients with low NRM and PK, we postulated clinically relevant Bu-AUC to be the defining set variable in a receiver operating characteristic (ROC)-curve model plotting Bu-AUC sensitivity versus 1-specificity to discriminate between patients with and without high NRM with special consideration of the respective area under the ROC (AUROC). The AUROC values illustrate the strength of a discriminating marker; that is, the better a diagnostic marker can discriminate between two conditions, the closer its AUROC value is to 1. The optimal cut-off point for Bu-AUC levels to discriminate for NRM was calculated using Youden’s index (Y)—Y = sensitivity + specificity − 1—because this method can be applied to find the optimal cut-off value with the highest sensitivity and specificity when there is no particular requirement for sensitivity and/or specificity. Statistical significance cut-off was determined by a *p* value ≤ 0.05. The softwares used to perform the statistical analysis were SPSS (version 22; IBM, Chicago, IL, USA) and STATA SE (version 15; StataCorp LLC, College Station, TX, USA).

## Results

### Patients’ baseline characteristics

A total of 658 patients received an allo-HCT between January 2013 and December 2020 at the University Hospital of Basel, of whom 376 had a conditioning regimen containing Bu and 300 were transplanted for a myeloid hematological neoplasm with Bu-PK available. Of these 300 patients, 253 were given Bu-4 and 47 Bu-1; 125 patients received CyBu or BuCy, 94 FluBu2, 49 FluBu4 and 32 TBF. The median age of patients was 56 years and 61% were male, 53% with a diagnosis of AML, 39% of MDS/MPN and 5% of CML. Regarding the disease stage, 2/3 were in complete remission or chronic phase (CML) and 30% without remission (5% were in second complete remission or never treated). Almost all patient received a transplant from peripheral blood (91%), 99 (33%) received a transplant from an HLA-identical sibling, 178 (60%) from a matched unrelated donor and 23 (8%) were haploidentical. About 18% had a previous allo-HCT (27 patients) or autologous HCT (26 patients). Patients with AML showed significantly lower-AUC values than MDS/MPN or CML patients. Regarding conditioning regimens, we show statistically higher AUC with BuCy (32% of patients in high-AUC) compared to the other regimens (8% with CyBu, 10% with FluBu4, 16% with TBF and 18% with Flubu2), whereas CyBu and FluBu4 had more often their PK under the AUC-target range (50% and 55%, respectively). In multivariate analysis models, underlying disease (*p* = 0.77) and conditioning regimen (*p* = 0.16) did not show any significant differences. Detailed baseline characteristics according to AUC are shown in Table 1.

**Table 1:** Patientsʼ baseline characteristcs according to Bu-AUC.

Baseline characteristics	Lower-AUC (*n* = 121)	In range AUC (*n* = 138)	Higher-AUC (*n* = 41)	*p* value
Age (median, yrs; range)	51 (18–74)	59 (24–72)	58 (24–73)	0.51
Patient gender (male, %)	68 (37)	93 (51)	21 (12)	0.08
Donor gender (male, %)	71 (39)	88 (49)	21 (12)	0.33
Donor/recipient gender				
Female/male (*n*, %)	28 (44)	27 (42)	9 (14)	0.13
Disease				**0.02**
AML (*n*, %)	80 (47)	63 (37)	26 (15)	
MDS/MPN (*n*, %)	37 (32)	66 (57)	13 (11)	
CML (*n*, %)	4 (27)	9 (60)	2 (13)	
Disease status at HCT				0.14
CR or chronic phase (*n*, %)	77 (43)	80 (44)	24 (13)	
2. CR or never treated (*n*, %)	2 (11)	12 (67)	4 (22)	
No CR (*n*, %)	42 (42)	46 (46)	13 (13)	
Prior HCT				
Allogeneic (*n*, %)	9 (33)	16 (59)	2 (7)	0.31
Autologous (*n*, %)	11 (42)	9 (35)	6 (23)	0.26
Stem cell source				
Peripheral blood (*n*, %)	107 (39)	131 (48)	35 (85)	0.08
Donor				
HLA-identical sibling (*n*, %)	31 (31)	50 (51)	18 (18)	0.15
HLA-matched unrelated (*n*, %)	78 (44)	80 (45)	20 (11)	
Haploidentical (*n*, %)	12 (52)	8 (35)	3 (13)	
Bu-conditioning regimen				**<0.01**
BuCy (*n*, %)	6 (32)	7 (37)	6 (32)	
CyBu (*n*, %)	53 (50)	45 (43)	8 (8)	
FluBu2 or FluBu3 (*n*, %)	21 (22)	56 (60)	17 (18)	
FluBu4 (*n*, %)	27 (55)	17 (35)	5 (10)	
Busulfan Thiotepa (*n*, %)	14 (44)	13 (41)	5 (16)	
Bu-administration				
4×/day (*n*, %)	90 (36)	127 (50)	36 (14)	**<0.01**
1×/day (*n*, %)	31 (66)	11 (23)	5 (11)	
Busulfan clearance in ml/min (IQR)	4.0 (3.8–4.5)	3.0 (2.7–3.3)	2.2 (2.0–2.3)	**<0.01**

Statistically significant *p*≤0.05 values are in bold.

*AUC* area under the curve, *AML* acute myeloid leukemia, *MDS* myelodysplastic syndrom, *MPN* myeloproliferative neoplasia, *CML* chronic myeloid leukemia, *CR* complete remission, *HCT* hematopoietic cell transplantation, *BuCy* busulfan-cyclophosphamide, *CyBu* cyclophosphamide-busulfan, *FluBu2* fludarabine-busulfan 2 days, *FluBu4* fludarabine-busulfan 4 days, *TBF* thiopetha-busulfan-fludarabine, *IQR* interquartile range.

### Busulfan pharmacokinetics

Regarding pharmacokinetics, median Bu-AUC was 1008 µmol/l*min (range 489–2664) in Bu-4 and 4217 µmol/l*min (range 2650 to 7764) for Bu-1; median Css 696 ng/mL in Bu-4 and 721 ng/mL in Bu-1 and cumulative AUC was 16231 µmol/l*min. The target range was reached in 138 (46%) patients, 121 (40%) were in low-AUC and 41 (14%) in high-AUC. The dosing of Bu at the second day of application was changed according to Bu-PK in 60 (20%) patients, 43 patients receiving an increased dose and 17 a decreased dose; 102 were not in target range but did receive their initial dose without modification, either because the difference from the AUC-target was too marginal (less than ±25% difference from target AUC) or because of ongoing relevant hepatic toxicity at time of Bu measurement (5 patients). Bu median clearance of all patient was 3.35 ml/min/kg, as expected it significantly decreased with high-PK values (4.0 ml/min/kg in high-AUC, 3.0 in target range and 2.2 ml/min/kg in low-AUC, *p* < 0.01).

### Correlation Bu-PK and toxicities

A total of 213 (71%) patients had at least one organ toxicity, mostly hepatic (*n* = 46, 15%; 2 patients fulfilling the SOS criteria) and/or renal (*n* = 40, 13%). There was no significant difference regarding the hepatic toxicity among the three groups (*n* = 21 (17%) in low-AUC, *n* = 16 (12%) in AUC-target and *n* = 9 (22%) in high-AUC; *p* = 0.20). Proved infectious complications were identified in a total of 160 (53%) patients, with a tendency of higher prevalence according to Bu-PK (49%, 54% and 66% in low-, in range and high-AUC respectively), thus not reaching statistical significance (*p* = 0.17). Viral and fungal infections significantly increased according to Bu-PK, as 17% of patients in high-AUC had a viral infection as compared to 8% in low-AUC (*p* = 0.03). 20% in the high-AUC had a fungal infection compared to 8% in low-AUC (*p* = 0.01). Bacterial infections slightly increased according to Bu-PK, without significant differences among the groups (*p* = 0.60). Other organ toxicities (renal, neurological, cardiac, pulmonary, dermatologic, MOF) and mucositis prevalence did not reach statistical difference, although we see a trend of increased incidence in almost all toxicities with higher-AUC (see Table 2). Incidence and severity of aGvHD and cGvHD were similar among the groups (*p* = 0.64 and 0.27, respectively). Time to engraftment was also similar according to Bu-PK (16, 18, and 17 days in low-AUC, target range and high-AUC respectively, *p* = 0.61).

**Table 2:** Outcomes according to busulfan pharmacokinetics (area under the curve).

Outcomes	Lower-AUC (*n* = 121)	In range AUC (*n* = 138)	Higher-AUC (*n* = 41)	*p* value (univariate)
Engraftment (median, days; range)	16 (11–67)	18 (11–90)	17 (8–26)	0.61
Acute GvHD ≥grade II (*n*, %)	32 (38)	38 (46)	14 (34)	0.64
aGvHD grade II (*n*, %)	19 (43)	16 (36)	9 (21)	0.54
aGvHD grade III (*n*, %)	10 (35)	14 (48)	5 (17)	
aGvHD grade IV (*n*, %)	3 (27)	8 (73)	0	
Chronic GvHD (*n*, %)	39 (32)	58 (42)	15 (37)	0.27
Toxicity, any (*n*, %)	85 (70)	94 (68)	34 (83)	0.12
Hepatic (*n*, %)	21 (17)	16 (12)	9 (22)	0.20
Mucositis grade ≥ III (*n*, %)	38 (31)	39 (28)	19 (46)	0.09
SOS/VOD (*n*, %)	1 (0.8)	1 (0.8)	0	0.85
Proven infection, any (*n*, %)	59 (49)	74 (54)	27 (66)	0.17
Bacterial infection (*n*, %)	43 (36)	57 (41)	15 (37)	0.62
Viral infection (*n*, %)	21 (17)	35 (25)	15 (37)	**0.03**
Fungal infection (*n*, %)	10 (8)	7 (5)	8 (20)	**0.01**
Neurological (*n*, %)	5 (4)	9 (6.5)	1 (2.4)	0.49
Pulmonary (*n*, %)	2 (1.5)	5 (3.5)	2 (5)	0.49
Cardiac (*n*, %)	3 (2.5)	5 (3.5)	2 (5)	0.74
Hemorrhagic cystitis (*n*, %)	5 (4)	10 (7)	3 (7)	0.53
Renal (*n*, %)	16 (13)	20 (15)	4 (10)	0.74
Dermatological (*n*, %)	11 (9)	11 (8)	4 (10)	0.92
Multiple organ failure (*n*, %)	1 (0.8)	2 (1.4)	0	0.69
Causes of death				0.21
Relapse (*n*, %)	19 (46)	25 (42)	10 (18)	
aGvHD (*n*, %)	7 (28)	14 (56)	4 (16)	
Infection (*n*, %)	1 (9)	7 (64)	3 (27)	
SOS/VOD (*n*, %)	0	1 (100)	0	
Other (*n*, %)	3 (25)	7 (58)	2 (1)	
Long-term outcomes (at 2 years)				
Overall survival (%, 95% CI)	67 (56–78)	60 (51–69)	52 (43–61)	0.06
NRM (%, 95% CI)	5 (2–13)	20 (14–29)	17 (8–39)	**<0.01**
Relapse (%, 95% CI)	46 (37–59)	30 (23–40)	41 (26–63)	0.28
GRFS (%, 95% CI)	28 (17–39)	25 (13–33)	18 (2–32)	0.28

Statistically significant *p*≤0.05 values are in bold.

*AUC* area under the curve, *aGvHD* acute graft-versus-host-disease, *cGvHD* chronic graft-versus-host-disease, *NRM* non-relapse mortality, *GRFS* graft-versus-host-disease-free-relapse-free-survival, *SOS* Sinusoidal obstruction syndrome, *VOD* veno-occlusive disease.

### Correlation BU-PK and long-term outcomes

**Fig. 1:**
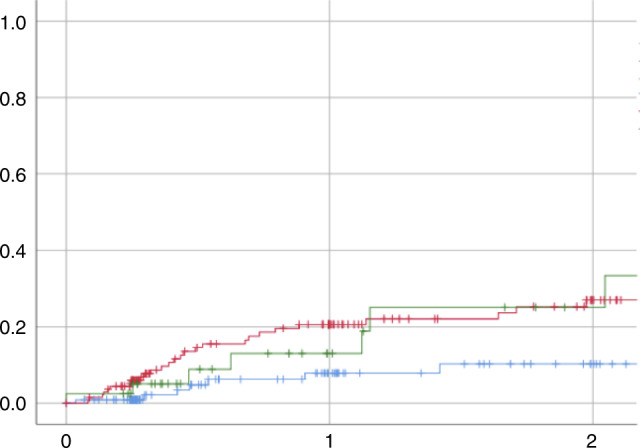
Non-relapse mortality according to Bu-PK. (Green Bu-PK in target range, RED BU-PK above target range, blue Bu-PK under target range).

**Fig. 2:**
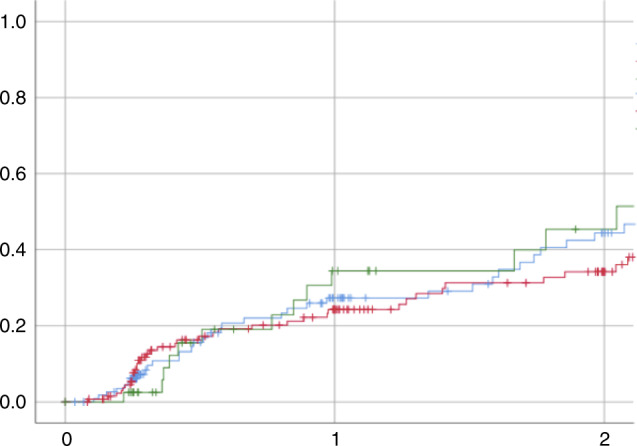
Relapse according to Bu-PK. (Green Bu-PK in target range, RED BU-PK above target range, blue Bu-PK under target range).

Long-term outcomes showed significant differences between the three groups. We showed a cumulative incidence for NRM at 2 years of 5 (2–13)% in low-AUC, compared to 20 (14–29)% in range AUC and 17 (8–39)% in high-AUC (*p* < 0.01, Fig. 1); causes of death did not differ (*p* = 0.21, Table 2). In multivariate analysis, relative risk (RR) of death in high-AUC was 1.9 (1.1–3.5; *p* = 0.02) compared to low-AUC; 1.62 (1.08–2.43, *p* = 0.02) in patients with advanced disease compared to CR; and 1.55 (1.01–2.38, *p* = 0.04) in HLA-matched unrelated compared to HLA-matched related transplants. A total of 94 (31%) were in relapse at 2 years, without any significant difference among the groups with a similar cumulative incidence of 46 (37–59)% in low-AUC, 30 (23–40)% in range and 41 (26–63)% in higher-AUC (*p* = 0.28) (Fig. 2). Univariate and multivariate analysis per outcome of interest is presented in Table 3. A separate analysis was run in the patients without dose adjustment (*n* = 240), showing similar results to the entire cohort. We do not have sufficient data to claim that the dose adjustment is ineffective but stress the fact that AUC performed on day 1 is most significantly associated with NRM.

**Table 3:** Multivariate analysis per outcome of interest.

	Univariate			Multivariate^a^		
Outcome per interest	Lower-AUC	In range AUC	Higher-AUC	Lower-AUC	In range AUC	Higher-AUC
Overall survival	1	1.4 (0.9–2.2) *p* = 0.15	1.7 (0.9–3.1) ***p*** = **0.06**	1	1.4 (0.9–2.2) *p* = 0.14	1.9 (1.1–3.5) ***p*** = **0.02**
NRM	1	4.0 (1.5–10.5) ***p*** = **0.04**	4.4 (1.4–13.5) ***p*** < **0.01**	1	3.9 (1.5–10.5) ***p*** = **0.05**	4.8 (1.6–14.7) ***p*** < **0.01**
Relapse	1	0.9 (0.6–1.4) *p* = 0.62	1.2 (0.7–2.2) *p* = 0.56	1	0.9 (0.6–1.4) *p* = 0.60	1.2 (0.6–2.1) *p* = 0.61
GRFS	1	1.2 (0.9–1.7) *p* = 0.18	1.3 (0.8–2.0) *p* = 0.18	1	1.2 (0.9–1.7) *p* = 0.14	1.5 (0.9–2.2) *p* = 0.09

Statistically significant *p*≤0.05 values are in bold.

*AUC* area under the curve, *NRM* non-relapse mortality, *GRFS* graft-versus-host-disease-free-relapse-free-survival, *SOS* Sinusoidal obstruction syndrome.

^a^Adjusted for disease stage and donor type.

**Fig. 3:**
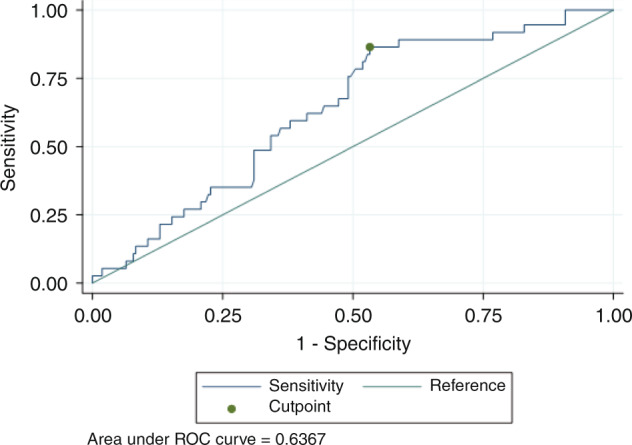
Bu-AUC cut-off range associated with lower NRM (ROC-curve).

When looking at the ROC curves, the empirical optimal cut-off in Bu-AUC associated with low NRM was 969 µmol/l*min (ROC AUC 0.67, sensitivity 0.86 and specificity 0.47) for Bu-4 (Fig. 3); the analysis was not done in Bu-1 population as the sample size was not sufficiently large.

### Pharmacokinetics and outcomes in bucy versus cybu

Given the difference of PK in BuCy versus CyBu (32% versus 50% in low-AUC, 37% versus 43% in target and 37% versus 8% in high-AUC, *p* < 0.01), we compared the toxicity and outcomes of the two regimens, even though it was not the primary endpoints of the study. Mean AUC was 918 (IQR 799–1157) µmol/l*min and Bu clearance was 3.5 (IQR 2.8–4.1) ml/min in CyBu, and mean AUC was 1161 (IQR 857–1364) µmol/l*min and mean clearance 2.8 (IQR 2.4–3.8) ml/min in BuCy (*p* = 0.025 for comparisons of clearance and AUC). As there was no patient receiving Bu 1×/d in BuCy, the comparison is in patients receiving Bu 4×/d only (114 patients). The only difference was seen in pulmonary toxicity, present in two patients (11%) in BuCy and one patient (1%) in CyBu (*p* = 0.01), but the low number of cases makes it difficult to draw a conclusion. Long-term outcomes at 2 years showed a tendency of worse outcome with slightly better survival (69% vs. 62%) and similar NRM (11% vs. 9%) with BuCy versus CyBu, not statistically different.

### Comparison Bu-4 versus Bu-1

Pharmacokinetics did differ significantly according to the number of doses given per day. Ninety (36%) patients were in low-AUC, 127 (50%) in range and 36 (14%) in high-AUC with Bu-4. Comparatively, more than half of the patients (66%) were in low-AUC and only 11% were in high-AUC with Bu-1 (*p* < 0.01). The median clearance of Bu did not show any significant difference though the tendency of higher clearance in Bu-1 is seen (3.22 ml/min/kg in Bu-4 versus 4.81 ml/min/kg in Bu-1, *p* = 0.23). The prevalence of dose adaptation was similar in the two groups. As for toxicities, the only significant difference between Bu-1 and Bu-4 was the incidence of mucositis, 29% of patients treated with Bu-4, compared to 51% when Bu-1 was given (*p* = 0.02). The incidences of aGvHD, cGvHD, overall toxicities, and all mentioned toxicities taken separately did not differ significantly among groups. We did not look at the long-term outcomes for this comparison as the follow-up period is too short for the patients receiving Bu 1×/d.

## Discussion

This is a retrospective study analyzing the association between busulfan dosing, pharmacokinetic target range and organ toxicities in patients with myeloid malignancies receiving Bu as part as their conditioning regimen before allogeneic stem cell transplantation. In patients with high-AUC, we show higher incidences of viral and fungal infections, a higher NRM and a higher relative risk of death without impact on relapse. The optimal AUC cut-off range with a significant reduction in NRM was 969 µmol/l*min. Bu-PK also differed when Bu was given once daily versus four times per day, with a higher prevalence of low-AUC but a higher incidence of mucositis.

Bu is widely used as chemotherapeutic drug for myeloablative or non-myeloablative conditioning regimen before allo-HCT. About 60% of all patients transplanted between 2013 and 2020 were conditioned with Bu, almost all of them to treat myeloid neoplasms (i.e., 95%). Bu PK dosing started in the 2000’s and introduced as TDM in the following years, allowing an important reduction of hepatic toxicity and the SOS, as the incidence of SOS has been reduced from about 15 to 3% in <10 years [4, 18]. Our target-AUC was reached in only 46% of the time, but we used narrower target limits than others (i.e., 800–1500 μmol/l*min [23], 900–1520 μmol/l*min [26]). Our busulfan median clearance (3.35 ml/min/kg) was within the higher limits of the normal range described in the literature [25], which explains why we have a high percentage of patients in the lower-AUC group. Bu-AUC performed on day 1 was significantly associated with NRM in all patients and also in those without dose adjustment. The group with dose adjustment was not large enough to study these effects (only 17 patients with a dose reduction).

The optimal target dose of Bu to permit a sufficient myeloablation with acceptable toxicity is still controversial and differs among studies; moreover, most of its clearance has been studied with BuCy, very few with FluBu and none with CyBu (but it has been accepted to use the same target dose with all conditioning regimens). With Bu-4, Andersson et al. describe a target AUC of <1200 μmol/l to prevent significant hepatic toxicity, with a serious increase of SOS and neurological side effects with an AUC > 1500 μmol/l*min [23], even though the effect on SOS was not confirmed in another study [15]. With Bu-1, Esteves et al. establish a target AUC < 5000 μmol/l*min [30]. An important multicenter retrospective study conducted in children defined a new optimal exposure AUC of 1225 to 1575 μmol/l*min with lower event-free survival as compared to children out of this range, particularly because of lower relapse probability at 2.5 years [31]; this higher target range is probably not applicable to adults because of toxicity.

The impact of hepatic toxicity with high-AUC values was not significant in our study (already shown by other authors [15]) and we had a very low SOS incidence (only 2 patients, 0.7%) comparing to the literature, where it’s described as occurring in about 3 to 5% with highly myeloablative conditioning [14, 18]. In our study about 30% of our patients received FluBu2, being non-myeloablative and also less toxic.

Regarding the other toxicities, very few studies have described extended and detailed impact of Bu-PK on organ toxicities, most of them were restricted to the central nervous system and the liver. Our results on organ toxicity according to Bu given 1×/d vs. 4×/d are consistent with previous studies comparing Bu given twice versus once daily, showing no significant difference on toxicity despite higher plasma concentration times when given 1×/d [20]. One study did show a significant difference on mucositis, hepatotoxicity and gastro-intestinal toxicity according to Bu-PK, although their population was limited to patients with CML [26]. It remains unclear why the patients receiving Bu-1 have a higher mucositis rate but a lower Bu-PK. It will be interesting to further look at long-term outcomes in these two populations (Bu-4 versus Bu-1), Bu-1 should benefit the patients at long-term with lower PK values. To our knowledge, we are the first study to describe the comparison of Bu-PK according to Bu-1 versus Bu-4 in adult patients, most of the previous studies were done in children [32, 33].

Esteves et al. had shown that a higher dose of Bu would not reduce relapse rates, similar to our results [30]. Another study did show a difference on relapse rate and aGvHD linked to Bu-AUC, although acknowledging the possible role of HLA incompatibility in their results [34]. Low-AUC is feared because of possible higher relapse rate, but we found a lower overall mortality with lower AUC-range at 2 years. A Japanese study with AML and MDS/MPN patients conditioned with FluBu4 (Bu given once daily) showed higher NRM and lower overall survival at 2 years in patients with high-AUC (i.e., Bu-AUC > 5000 μmol/l*min) but nevertheless lower relapse rate when the second AUC (day + 4) was above 6000 μmol/l*min, also suggesting that the NRM with high-AUC values counterbalance the higher relapse rate [35]. A recently published study analyzed long-term outcome of myeloablative FluBu in older patients, using Bu 80 mg/m^2^ on days −13 and −12 and then addition of Flu 40 mg/m^2^ from days −6 to −3 with a NRM of 22% at 3 years (14% vs. 29% according to HCT-CI score). This could be an alternative for older patients who could nonetheless benefit from an allo-HCT [36].

The impact of different conditioning regimens on Bu-PK has never been fully studied and centers use the same target range for all types of conditioning with Bu. Kikuchi et al. describe a possible effect of Fludarabine affecting the metabolism of Bu when given before, with higher AUC as compared to BuCy [1], but these results are not in agreement with other studies. The differences in Bu_PK seen in this study here, seem to benefit CyBu over BuCy. In a previous randomized trial analyzing the hepatic toxicity of CyBu versus BuCy, we found that CyBu had a lower NRM even though pharmacokinetics did not differ according to conditioning regimen [21], but the population was smaller and differed slightly because it was not limited to myeloid neoplasm. Hassan et al. conducted a study on 36 patients who received BuCy and showed that a short time interval <24 h between Bu and Cy could lower the Cy clearance and therefore increase incidence of hepatic toxicity and mucositis [22], this time interval was respected in our study.

We acknowledge the limitation of a retrospective study restricted to myeloid neoplasm and with heterogeneity of conditioning regimens and Bu-administration. The low number of patients receiving Bu-1 compared to Bu-4 (47 versus 253) is a limitation due to lack of follow-up data. Nonetheless, with an important cohort size of 300 patients, these results are the first step to future randomized trials for a possible reduction of Bu AUC-target range as the long-term outcomes seem to have an impressive impact. Bu-1 having significantly reduced Bu-AUC, long-term outcomes of patients receiving this regimen instead of Bu-4 will be of interest to analyze. As high-AUC seems to show a higher incidence of fungal and viral infections, this could be a future focus for prophylaxis and intensive screening. In conclusion, low-AUC BU-PK seems of benefit regarding NRM and survival.

## Supplementary information

Supplementary Table 1

## Data Availability

The datasets generated during and/or analysed during the current study are available from the corresponding author on reasonable request.

## References

[1] Kikuchi T, Mori T, Ohwada C, Onoda M, Shimizu H, Yokoyama H (2020). Pharmacokinetics of intravenous busulfan as condition for hematopoietic stem cell transplantation: comparison between combinations with cyclophosphamide and fludarabine. Int J Hematol..

[2] Vassal G, Hartmann O, Benhamou E (1990). Busulfan and veno-occlusive disease of the liver. Ann Intern Med.

[3] Tsakiris DA, Tichelli A (2009). Thrombotic complications after haematopoietic stem cell transplantation: early and late effects. Best Pract Res Clin Haematol.

[4] Bleyzac N, Souillet G, Magron P, Janoly A, Martin P, Bertrand Y (2001). Improved clinical outcome of paediatric bone marrow recipients using a test dose and Bayesian pharmacokinetic individualization of busulfan dosage regimens. Bone Marrow Transplant.

[5] Copelan EA, Bechtel TP, Avalos BR, Elder PJ, Ezzone SA, Scholl MD (2001). Busulfan levels are influenced by prior treatment and are associated with hepatic veno-occlusive disease and early mortality but not with delayed complications following marrow transplantation. Bone Marrow Transplant.

[6] Masson E, Zamboni WC (1997). Pharmacokinetic optimisation of cancer chemotherapy. Effect on outcomes. Clin Pharmacokinet.

[7] DeLeve LD, Wang X (2000). Role of oxidative stress and glutathione in busulfan toxicity in cultured murine hepatocytes. Pharmacology..

[8] Hassan Z, Hellström-Lindberg E, Alsadi S, Edgren M, Hägglund H, Hassan M (2002). The effect of modulation of glutathione cellular content on busulphan-induced cytotoxicity on hematopoietic cells in vitro and in vivo. Bone Marrow Transplant.

[9] Ansari M, Curtis PH-D, Uppugunduri CRS, Rezgui MA, Nava T, Mlakar V (2017). GSTA1 diplotypes affect busulfan clearance and toxicity in children undergoing allogeneic hematopoietic stem cell transplantation: a multicenter study. Oncotarget..

[10] Ansari M, Huezo-Diaz P, Rezgui MA, Marktel S, Duval M, Bittencourt H (2016). Influence of glutathione S-transferase gene polymorphisms on busulfan pharmacokinetics and outcome of hematopoietic stem-cell transplantation in thalassemia pediatric patients. Bone Marrow Transplant.

[11] Kim MG, Kwak A, Choi B, Ji E, Oh JM, Kim K (2019). Effect of glutathione S-transferase genetic polymorphisms on busulfan pharmacokinetics and veno-occlusive disease in hematopoietic stem cell transplantation: a meta-analysis. Basic Clin Pharmacol Toxicol.

[12] Nava T, Kassir N, Rezgui MA, Uppugunduri CRS, Huezo-Diaz Curtis P, Duval M (2018). Incorporation of GSTA1 genetic variations into a population pharmacokinetic model for IV busulfan in paediatric hematopoietic stem cell transplantation. Br J Clin Pharmacol.

[13] Myers AL, Kawedia JD, Champlin RE, Kramer MA, Nieto Y, Ghose R (2017). Clarifying busulfan metabolism and drug interactions to support new therapeutic drug monitoring strategies: a comprehensive review. Expert Opin Drug Metab Toxicol.

[14] Kashyap A, Wingard J, Cagnoni P, Roy J, Tarantolo S, Hu W (2002). Intravenous versus oral busulfan as part of a busulfan/cyclophosphamide preparative regimen for allogeneic hematopoietic stem cell transplantation: decreased incidence of hepatic venoocclusive disease (HVOD), HVOD-related mortality, and overall 100-day mortality. Biol Blood Marrow Transplant.

[15] Andersson BS, Kashyap A, Gian V, Wingard JR, Fernandez H, Cagnoni PJ (2002). Conditioning therapy with intravenous busulfan and cyclophosphamide (IV BuCy2) for hematologic malignancies prior to allogeneic stem cell transplantation: a phase II study. Biol Blood Marrow Transplant.

[16] Hassan Z, Nilsson C, Hassan M (1998). Liposomal busulphan: bioavailability and effect on bone marrow in mice. Bone Marrow Transplant.

[17] Wang Y, Kato K, Le Gallo C, Armstrong E, Rock E, Wang X (2015). Dosing algorithm revisit for busulfan following IV infusion. Cancer Chemother Pharmacol.

[18] El-Serafi I, Remberger M, Ringdèn O, Törlén J, Sundin M, Björklund A (2019). Reduced risk of sinusoidal obstruction syndrome of the liver after busulfan-cyclophosphamide conditioning prior to allogeneic hematopoietic stem cell transplantation. Clin Transl Sci..

[19] Kletzel M, Jacobsohn D, Duerst R (2006). Pharmacokinetics of a test dose of intravenous busulfan guide dose modifications to achieve an optimal area under the curve of a single daily dose of intravenous busulfan in children undergoing a reduced-intensity conditioning regimen with hematopoietic stem cell transplantation. Biol Blood Marrow Transplant.

[20] Fernandez HF, Tran HT, Albrecht F, Lennon S, Caldera H, Goodman MS (2002). Evaluation of safety and pharmacokinetics of administering intravenous busulfan in a twice-daily or daily schedule to patients with advanced hematologic malignant disease undergoing stem cell transplantation. Biol Blood Marrow Transplant.

[21] Seydoux C, Medinger M, Gerull S, Halter J, Heim D, Chalandon Y (2020). Busulfan-cyclophosphamide versus cyclophosphamide-busulfan as conditioning regimen before allogeneic hematopoietic cell transplantation: a prospective randomized trial. Ann Hematol.

[22] Hassan M, Ljungman P, Ringdén O, Hassan Z, Oberg G, Nilsson C (2000). The effect of busulphan on the pharmacokinetics of cyclophosphamide and its 4-hydroxy metabolite: time interval influence on therapeutic efficacy and therapy-related toxicity. Bone Marrow Transplant.

[23] Andersson BS, Madden T, Tran HT, Hu WW, Blume KG, Chow DS (2000). Acute safety and pharmacokinetics of intravenous busulfan when used with oral busulfan and cyclophosphamide as pretransplantation conditioning therapy: a phase I study. Biol Blood Marrow Transplant.

[24] Yeh RF, Pawlikowski MA, Blough DK, McDonald GB, O’Donnell PV, Rezvani A (2012). Accurate targeting of daily intravenous busulfan with 8-hour blood sampling in hospitalized adult hematopoietic cell transplant recipients. Biol Blood Marrow Transplant.

[25] Slattery JT, Risler LJ (1998). Therapeutic monitoring of busulfan in hematopoietic stem cell transplantation. Ther Drug Monit.

[26] Andersson BS, Thall PF, Madden T, Couriel D, Wang X, Tran HT (2002). Busulfan systemic exposure relative to regimen-related toxicity and acute graft-versus-host disease: defining a therapeutic window for i.v. BuCy2 in chronic myelogenous leukemia. Biol Blood Marrow Transplant.

[27] Mohty M, Malard F, Abecassis M, Aerts E, Alaskar AS, Aljurf M (2016). Revised diagnosis and severity criteria for sinusoidal obstruction syndrome/veno-occlusive disease in adult patients: a new classification from the European Society for Blood and Marrow Transplantation. Bone Marrow Transplant.

[28] Przepiorka D, Weisdorf D, Martin P, Klingemann HG, Beatty P, Hows J (1995). 1994 Consensus Conference on Acute GVHD Grading. Bone Marrow Transplant.

[29] Binkert L, Medinger M, Halter JP, Heim D, Gerull S, Holbro A (2015). Lower dose anti-thymocyte globulin for GvHD prophylaxis results in improved survival after allogeneic stem cell transplantation. Bone Marrow Transplant.

[30] Esteves I, Santos FPS, Ribeiro AAF, Seber A, Sugawara EK, Sobrinho JJ (2020). Targeted-dose of busulfan: Higher risk of sinusoidal obstructive syndrome observed with systemic exposure dose above 5000 µMol min. A historically controlled clinical trial. Hematol Oncol..

[31] Bartelink IH, Lalmohamed A, van Reij EML, Dvorak CC, Savic RM, Zwaveling J (2016). Association of busulfan exposure with survival and toxicity after haemopoietic cell transplantation in children and young adults: a multicentre, retrospective cohort analysis. Lancet Haematol.

[32] Bartelink IH, Bredius RGM, Belitser SV, Suttorp MM, Bierings M, Knibbe CAJ (2009). Association between busulfan exposure and outcome in children receiving intravenous busulfan before hematologic stem cell transplantation. Biol Blood Marrow Transplant.

[33] Philippe M, Neely M, Rushing T, Bertrand Y, Bleyzac N, Goutelle S (2019). Maximal concentration of intravenous busulfan as a determinant of veno-occlusive disease: a pharmacokinetic-pharmacodynamic analysis in 293 hematopoietic stem cell transplanted children. Bone Marrow Transplant.

[34] Slattery JT, Sanders JE, Buckner CD, Schaffer RL, Lambert KW, Langer FP (1995). Graft-rejection and toxicity following bone marrow transplantation in relation to busulfan pharmacokinetics. Bone Marrow Transplant.

[35] Ohwada C, Yamazaki S, Shono K, Kayamori K, Hino Y, Oshima-Hasegawa N (2021). Pharmacokinetically guided, once-daily intravenous busulfan in combination with fludarabine for elderly AML/MDS patients as a conditioning regimen for allogeneic stem cell transplantation. Int J Hematol.

[36] Mehta RS, Bassett R, Chen J, Valdez BC, Kawedia J, Alousi AM (2021). Myeloablative fractionated busulfan with fludarabine in older patients: long term disease-specific outcomes of a prospective phase II clinical trial. Transplant Cell Ther.

