# Supplementary material for: Impact of busulfan pharmacokinetics on outcome in adult patients receiving an allogeneic hematopoietic cell transplantation
Source: Bone Marrow Transplant. 2022 Mar 31;57(6):903–10. doi: 10.1038/s41409-022-01641-6 (PMC9200635; doi:10.1038/s41409-022-01641-6)
Supplement: Supplementary file 1 — Supplementary Table 1 [file 41409_2022_1641_MOESM1_ESM.pdf]

**Chemotherapy**	**Day -7**	**Day -6**	**Day -5**	**Day -4**	**Day -3**	**Day -2**	**Day -1**	**Day 0**
**BuCy**	Bu	Bu	Bu	Bu	Cy 60mg/kg (1h)	Cy 60mg/kg (1h)		Allo-HCT
**CyBu**	Cy 60mg/kg (1h)	Cy 60mg/kg (1h)	Bu	Bu	Bu	Bu		Allo-HCT
**TBF**		Thiotepa 5mg/kg (2h)	Thiotepa 5mg/kg (2h)	Bu<br>Flu 50mg/m^2 (30min)	Bu<br>Flu 50mg/m^2 (30min)	Flu 50mg/m^2 (30min)		Allo-HCT
**FluBu4**		Bu<br>Flu 30mg/m^2 (30min)	Bu<br>Flu 30mg/m^2 (30min)	Bu<br>Flu 30mg/m^2 (30min)	Bu<br>Flu 30mg/m^2 (30min)	Flu 30mg/m^2 (30min)		Allo-HCT
**FluBu2**		Bu<br>Flu 30mg/m^2	Bu<br>Flu 30mg/m^2	Flu 30mg/m^2 (30min)	Flu 30mg/m^2 (30min)	Flu 30mg/m^2 (30min)		Allo-HCT

		(30min)	(30min)					
